# High frequency of DNA detection of *toxoplasma gondii* and zoonotic *Sarcocystis* spp. in ready-to-eat meat products purchased in Switzerland

**DOI:** 10.1016/j.fawpar.2025.e00301

**Published:** 2025-11-12

**Authors:** Z. Medici, N. Marreros, S. Molteni, M.C. Ferreira de Sousa, W. Basso, G. Moré, C.F. Frey

**Affiliations:** a Institute of Parasitology, Department of Infectious Diseases and Pathobiology, Vetsuisse Faculty, University of Bern, Länggassstrasse 122, 3012 Bern, Switzerland; b School of Agricultural, Forest and Food Sciences HAFL, Bern University of Applied Sciences, Länggasse 85, 3052, Zollikofen, Switzerland; c Department of Veterinary Medicine and Animal Sciences (DIVAS), University of Milan, Via dell'Università 6, 26900 Lodi, Italy

**Keywords:** Food safety, Magnetic capture DNA extraction, qPCR, Sequencing, Zoonosis, Cured ham

## Abstract

*Toxoplasma gondii* and *Sarcocystis* spp. are globally distributed, intracellular, cyst-forming coccidian parasites that infect a wide range of animal species, and humans. These protozoan parasites have indirect life cycles and can be transmitted to hosts through food sources, such as infected meat. Resulting infections may pose serious health risks, especially for immunocompromised individuals and developing foetuses. While their prevalence in Switzerland has been studied serologically and molecularly in live animals or after necropsy or slaughter, there are no studies on ready-to-eat (RTE) meat products. This study aimed to assess the presence of these parasites in RTE meat products sourced from Swiss supermarkets, retail stores and local butcher shops. A total of 201 RTE meat products consisting of pork, beef, poultry, game, and equine meat, as well as mixes thereof, were tested. Hundred-gram samples were homogenized, followed by a sequence-specific magnetic capture and real-time PCR for *T. gondii* DNA, and a crude DNA extraction and PCR – Sanger sequencing for *Sarcocystis* spp. For two zoonotic species (*S. suihominis* and *S. hominis*), additional PCRs were performed. Furthermore, the homogenates were analyzed for *Sarcocystis* spp. cysts by stereomicroscopy. Variables associated with the presence of these parasites were identified by multivariable LASSO regression. *Toxoplasma gondii* DNA was detected in 14.9 % (30/201) of the samples, while *Sarcocystis* spp. DNA was present in 58.2 % (117/201). Zoonotic *S. suihominis* DNA was found in 3.2 % (4/125) of the samples containing pork, and *S. hominis* DNA in 29.6 % (24/81) of the samples containing beef. No viable cysts were observed in any sample. The presence of *T. gondii* DNA was associated with the variables pork, salami-like products, and wild boar. *Sarcocystis* spp. DNA was positively associated with beef and salami-like products, and negatively with chicken and Swiss pork. *Sarcocystis hominis* was positively associated with Swiss beef. These findings reveal a notable rate of RTE meat products positive for zoonotic parasites, suggesting a potential public health risk. Further research is needed to evaluate their role in transmission to humans.

## Highlights

• 201 RTE meat products analyzed for *Toxoplasma gondii* and *Sarcocystis* spp. DNA.
• *T. gondii* DNA detected in 14.9 % and *Sarcocystis* spp. DNA in 58.2 % of samples.
• *S. hominis* found in 29.6 % of beef samples and *S. suihominis* in 3.2 % of pork samples.
• Pork had highest positivity for *T. gondii* and beef for *Sarcocystis* spp., respectively.
• Salami-type products had a higher positivity than ham-type products.

## Graphical abstract

**Figure:**
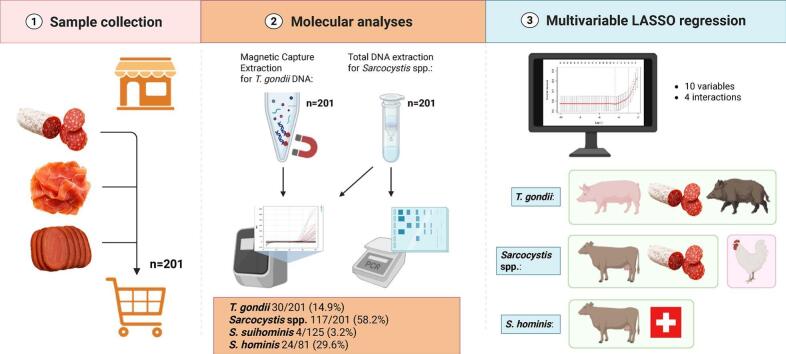

## 1 Introduction

*Toxoplasma gondii* and *Sarcocystis* spp. are obligate intracellular apicomplexan parasites, that are often transmitted through food or water and can pose health risks to humans (Devleesschauwer et al., 2017). These parasites are cyst-forming coccidia with relatively complex life cycles and intricate stages of development, involving definitive hosts that are responsible for the dissemination of oocysts or sporocysts into the environment, and a wide range of intermediate hosts, which act as living carriers for slow replicating stages of the parasite, i.e. tissue cysts filled with infectious bradyzoites. Definitive and intermediate hosts typically consist of predator and prey animals, respectively (Dubey, 2022; Dubey et al., 2016). Due to their impact on human health, trade and economic relevance, *T. gondii* and *Sarcocystis* spp. have been listed among the most important foodborne parasites globally (Devleesschauwer et al., 2017).

Felids are the only definitive hosts for *T. gondii*. Infection of animal intermediate hosts and humans can mainly occur via ingestion of oocyst-contaminated water or food, consumption of tissue cysts in raw or inadequately cooked meat, or vertical transmission of tachyzoites during pregnancy (Hill and Dubey, 2002). Although all three transmission routes are significant, their relative contribution in humans varies geographically depending on cultural culinary practices and environmental contamination levels with oocysts (Dubey, 2022). In Europe, consumption of raw or undercooked meat is considered as a major source of infection. Up to 60 % of *T. gondii* infections in pregnant women were assumed to be linked to the consumption of meat, especially beef, pork, and small ruminants (BIOHAZ, E. P. on B. H, et al., 2018; Cook et al., 2000). *Toxoplasma gondii* infection in humans is in most cases asymptomatic or presents with mild self-resolving symptoms like fever, myalgia, swollen lymph nodes, and fatigue. However, individuals with a weakened immune system, or healthy individuals infected by atypical strains may face severe and potentially life- threatening outcomes (Hill and Dubey, 2002; Sobanski et al., 2013). Other serious forms are congenital toxoplasmosis, acquired through primary maternal infection during pregnancy, possibly producing miscarriage, stillbirth, or severe congenital defects, and ocular toxoplasmosis, which can be acquired in utero as well as postnatally, causing chorioretinitis and visual impairment (Dubey, 2021; Pleyer et al., 2014). In animals most infections are asymptomatic. Nevertheless, *T. gondii* can cause severe illness in cats and dogs, including respiratory distress, hepatitis, neurological signs, ocular lesions, and even death. In livestock, particularly pigs and small ruminants, the infection can be also associated with abortion and other reproductive disorders (Dubey, 2022).

The genus *Sarcocystis* comprises over 200 species distributed worldwide, each with specific definitive and intermediate hosts. Humans, along with other omnivores and carnivores such as canids and felids, mostly act as definitive hosts and become infected by ingesting viable bradyzoites in muscle tissue cysts. To date, four *Sarcocystis* species are known to infect humans via consumption of infected meat: *S. suihominis* from pork, and *S. hominis*, *S. heydorni*, and *S. sigmoideus* from beef (Dubey, 2015; Moniot et al., 2025; Rubiola et al., 2024). Epidemiologic data on *Sarcocystis* infections in humans is limited. Prevalence of human intestinal sarcocystosis ranged from 1.1 to 10.4 % in European countries (Dubey et al., 2016; Poulsen and Stensvold, 2014). Infection in humans is mostly subclinical, but patients may experience severe stomachache, nausea, vomiting, diarrhea and even dyspnea within 24 h after ingestion. *Sarcocystis suihominis* infections tend to be more severe than those caused by *S. hominis*. The health impact of *S. heydorni* and *S. sigmoideus* remains unclear (Fayer et al., 2015; Moniot et al., 2025; Rosenthal, 2021). Predator animals acting as definitive hosts of *Sarcocystis* spp. are typically asymptomatic or may develop mild enteritis. Intermediate hosts' infections are generally asymptomatic and chronic (with presence of muscle cysts or sarcocysts). Rarely, intermediate hosts like pigs (*S. miescheriana, S. suihominis*) and cattle (*S. cruzi, S. hominis, S. rommeli/S. bovifelis, S. hirsuta, S. sigmoideus, S. heydorni*) exhibit more severe signs, including anorexia, anemia, weakness, dyspnea, muscle tremors, abortion, and even death, depending on the number of sporocysts ingested (Dubey et al., 2016). *Sarcocystis* spp. infection in livestock also has an economic importance in that carcasses with visible tissue cysts or discoloration known as eosinophilic myositis are unfit for human consumption (Dubey et al., 2016; Rubiola et al., 2021, Rubiola et al., 2024; Verordnung des EDI über die Hygiene beim Schlachten (VHyS), 2006).

Ready-to-eat (RTE) meat products, such as dried ham and cured sausages, are raw products intended for direct human consumption without the need for cooking or microbial inactivation. Thus, they may still harbor infective tissue cysts of *T. gondii* or *Sarcocystis* spp. and therefore pose a potential risk to consumers (Gomez-Samblas et al., 2015; Herrero et al., 2017). Regarding prevalence of parasites, previous studies on RTE meat products are very limited. Direct detection of *T. gondii* in commercially available meat products showed variable results and a comparison appears difficult because of the different sample types and methods employed. European studies found prevalences of 1.5–8.8 % for *T. gondii* in RTE meat products, whereas studies about these products in Brazil showed a prevalence of 15.6–17 % (Bayarri et al., 2012; Costa et al., 2018; Espindola et al., 2022; Gomez-Samblas et al., 2015; Sroka et al., 2019; Warnekulasuriya et al., 1998). *Sarcocystis* spp. in RTE meat products was investigated through direct methods in one study, yielding a prevalence of 62.5 % (Espindola et al., 2022). In Switzerland, the prevalence of these parasites has been studied serologically and molecularly in live animals or after necropsy or slaughter; however, there are no studies on RTE meat products (Basso et al., 2020, Basso et al., 2022; Berger-Schoch et al., 2011a; Moré et al., 2025).

The aim of this study was to determine how frequently DNA of *T. gondii* or *Sarcocystis* spp. (with particular attention to zoonotic *S. suihominis* and *S. hominis*) is detected in commercially available raw RTE meat products. Recognizing that the detection of DNA alone does not confirm the presence of viable or infectious parasites, homogenates were microscopically screened for sarcocysts and viability assessed by supravital staining. In addition, variables potentially associated with the presence of pathogen DNA were explored.

## 2 Material and methods

### 2.1 Samples

A total of 201 RTE meat products, each weighing more than 105 g, were purchased in Switzerland from November 2024 to April 2025. These samples were sourced from consumer-accessible shops, namely from all major supermarket chains (*n* = 161 samples), retail butchers (*n* = 17), local shops (*n* = 10), organic shops (n = 10), and farmer's markets (*n* = 3). The samples primarily consisted of pre-packaged raw cold cuts or cured meat assortments derived from pork, beef, equines, poultry, and game, and mixes of those species (Table 1). Of the total samples, 122 contained meat from animals raised in Switzerland, 74 meat from other European countries, six from non-European countries, and for two it was not possible to define the origin of the meat with certainty. Different types of processed meat were included and classified as salami-like (ground or mixed meat, e.g. salami, *chorizo,*e.g.) or ham-like (whole, e.g. cured ham, *coppa*, *mostbröckli*, *bresaola*,e.g.). A total of 36 samples were labelled as produced from organic farming (Table 1). Products without organic label were considered non-organic, even if they could partially include ingredients from organic farming. Samples were maintained at 4–8 °C no longer than one week before further processing.

**Table 1:** Distribution of ready-to-eat meat samples (*n* = 201), based on animal species, origin of the meat, product type, and organic farming labels.

Species	Total	Meat composition		Meat origin ^a^			Product type ^c^		Organic farming^d^
		Pure	Mixed	Swiss	EU	Other ^b^	Salami-like	Ham-like	
Pork	125	96	29	74	49	2	64	61	24
Beef	81	55	26	60	21	1	34	47	20
Horse	7	5	2	2	3	2	2	5	0
Poultry	15	11	4	8	7	0	12	3	0
Wild boar	4	0	4	0	4	0	4	0	0
Deer	3	0	3	2	1	2	3	0	0
Total^a^	201	167	34	122	74	6	85	116	36

a Does not equal sum of individual meat types due to products containing more than one type of meat (“mixed”) or origin. ^b^Specifically includes South America, USA, New Zealand and Australia. ^c^Products derived from ground meat classified as “salami-like”, whole cuts pieces classified as “ham-like”. ^d^Classification based on Swiss criteria.

### 2.2 Sample digestion, total and magnetic capture DNA extraction

For each sample, 100 g were weighed, cut, and placed into a Stomacher400 classic bag with sterile strainer/filter (Seward, UK). Where feasible, fat, and connective tissue were manually removed, although complete removal was often not possible due to the nature of the samples. The samples were homogenized in a Stomacher400 at 260 rpm for 2 min with 250 mL of lysis buffer (100 mM Tris-HCl pH 8.0, 5 mM EDTA pH 8.0, 0.2 % SDS, 200 mM NaCl) containing 10 mg of Proteinase K (Carl Roth, Switzerland). The homogenate was incubated on an orbital shaker overnight at 55 °C and 70 rpm.

**Fig. 1:**
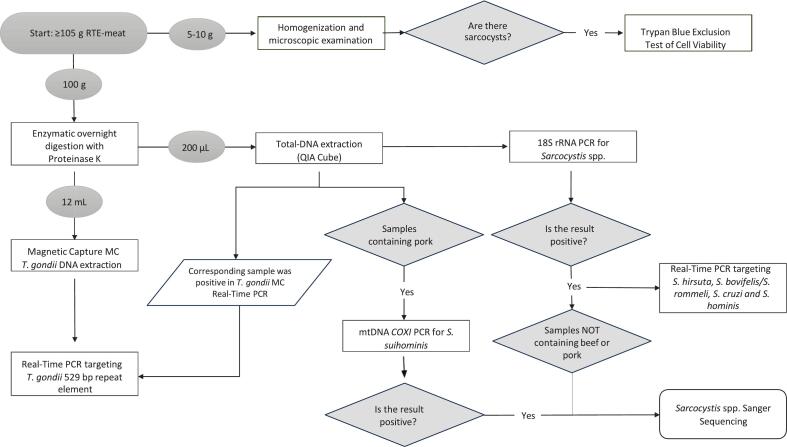
Workflow of molecular and microscopical analyses. Analyses applied to 201 samples of RTE meat products.

The following day, 50 mL of the lysate were transferred to a DNase free tube and centrifuged at 3500 ×*g* for 45 min. This allowed the removal of the fat layer and collection of the liquid-phase lysate. The lysate was divided in aliquots, to perform two separate DNA extractions (Fig. 1). The first one was a total-DNA extraction employing the DNeasy Blood & Tissue Kit in the QIAcube Connect device (QIAGEN GmbH, Hilden, Germany) on 200 μL of lysate.

The second extraction method targeted a highly conserved 529-bp repeat element of *T. gondii* via magnetic capture, essentially proceeding as previously described (Opsteegh et al., 2010). Briefly, 12 mL of the lysate were transferred to a DNase free 15 mL tube. Following thermal inactivation of Proteinase K at 100 °C for 10 min, the lysate was incubated with Pierce™ High-Capacity Streptavidin Agarose Resin (Thermo Fisher Scientific, USA) to remove native biotin. After centrifugation at 3500 ×*g* for 15 min, 10 mL of the resulting supernatant was collected and hybridized with 70-bp forward and reverse oligonucleotides (ToxCapF and ToxCapR) labelled at the 5’-OH end with a biotin molecule (Microsynth AG, Switzerland) (Table 2). These hybridized oligonucleotides were then captured using Dynabeads™ M-270 Streptavidin magnetic beads, enabling the magnetic isolation of the bound DNA via magnetic racks (Thermo Fisher Scientific, USA). The beads were washed four times with decreasing volumes of wash buffer (10, 5, 3, and 1.5 mL, respectively) and the DNA was eluted (release of DNA from the Dynabeads) in 50 μL of distilled water by thermal denaturation at 99 °C for 10 min and final separation using a magnetic rack.

**Table 2:** Oligonucleotides used in this study, indicating role and reference study.

Name	Sequence	Work conc.	Role	Method	Reference
Tox-CapF	5′-(Biotin TEG)- CTTGGAGCCA CAGAAGGGAC AGAAGTCGAA GGGGACTACA GACGCGATGC CGCTCCTCCA GCCGTCTTGG −3′	1 μM	Capture oligonucleotides	1	(Opsteegh et al., 2010)
Tox-CapR	5′-(Biotin TEG)- AAGCCTCCGA CTCTGTCTCC CTCGCCCTCT TCTCCACTCT TCAATTCTCT CCGCCATCAC CACGAGGAAA −3′	1 μM	Capture oligonucleotides	1	(Opsteegh et al., 2010)
Tox-9	5′- AGGAGAGATA TCAGGACTGT AG −3′	20 μM	Forward Primer	2	(Reischl et al., 2003)
Tox-11	5′- GCGTCGTCTC GTCTAGATCG −3′	20 μM	Reverse Primer	2	(Reischl et al., 2003)
Tox-HP1	5′-(FAM)- GAGTCGGAGA GGGAGAAGAT GTT -(BHQ1)-3′	10 μM	TaqMan Probe	2	(Pardo Gil et al., 2023; Reischl et al., 2003)
SarcoF	5′- CGCAAATTAC CCAATCCTGA −3′	100 μM	Forward Primer	3	(Moré et al., 2011)
SarcoR	5′- ATTTCTCATA AGGTGCAGGA g-3′	100 μM	Reverse Primer	3	(Moré et al., 2011)
SF8	5′- CTTGGAGCGG TATGGCTAAT −3’	100 μM	Forward Primer	4	(Gazzonis et al., 2019)
SR11	5′- GGAAGTGGGC AACAATGTAA TA −3′	100 μM	Reverse Primer	4	(Gazzonis et al., 2019)
SarcoRTF	5′- ACCCATCTAT ATTGGGATAA TACCGTTACT −3′	10 μM	Forward Primer	5	(Moré et al., 2013)
SarcoRTR	5′- AGGCAATAAG CCTGCTTCAA −3′	10 μM	Reverse Primer	5	(Moré et al., 2013)
ShirsutaRTR	5′- GCAACAATAA GCCTGCTTCA AA −3′	10 μM	Reverse Primer	5	(Moré et al., 2013)
Scruzi	5′-(RED)- ACCCATCTAT ATTGGGATAA TACCGTTACT -(BHQ1)-3′	10 μM	TaqMan Probe	5	(Moré et al., 2013)
Shirsuta	5′-(FAM)- CCTTCTAATG AGGGTGTGTA CTTGATGAA -(BHQ1)-3′	10 μM	TaqMan Probe	5	(Moré et al., 2013)
ShomLNA	5′-(Cy5)-TCT(+T)1(+A)TA(+T)AA(+T)GA(+T)TA(+T)TG(+A)A(+T)TGA-(BHQ2)-3′	10 μM	TaqMan Probe	5	(Moré et al., 2013)
SromLNA	5′-(HEX)-CTG(+A)TG(+A)CT(+T)TC(+A)GT(+A)GTCATt-(BHQ1)-3′	10 μM	TaqMan Probe	5	(Moré et al., 2013)

Ref. Method 1: magnetic capture DNA extraction for *T. gondii*; 2: *T. gondii* 529 bp repeat qPCR; 3: *Sarcocystis* spp. *18S* rRNA PCR; 4: *S. suihominis* specific *COI* mtDNA PCR; 5: multiplex PCR for *Sarcocystis* spp. in cattle.

DNA was maintained at −20° till further processing.

### 2.3 Real-time PCR targeting *Toxoplasma gondii* 529 bp highly repeated element

DNA of all samples obtained through sequence-specific magnetic capture extraction was tested for *T. gondii* using real-time PCR, as previously reported (Pardo Gil et al., 2023) (Table 2). Briefly, each 10 μL reaction contained 5 μL of 2× Mastermix (SensiFAST™ Probe NO-ROX Kit; Bioline Meridian Life Science, Memphis, USA), 0.25 μL each of forward (Tox9) and reverse (Tox11) primers, 0.1 μL of TaqMan hydrolysis probe (Tox-HP-1), 0.3 μL of P-dUTP, 0.1 μL of P-UDG, 2 or 4 μL of DNA template and nuclease-free water adjusting to the final volume. Each sample was tested in duplicate, with either 2 or 4 μL of DNA, and for each reaction, an additional reaction including an internal plasmid-based inhibition control was performed. Each PCR routine included a non-template control (4 μL ultrapure water), and a positive control (*T. gondii* RH-strain tachyzoites) (Pardo Gil et al., 2023). When inhibition was detected, the sample was retested using lower volumes of template DNA or a 1:10 dilution.

PCR was performed in the CFX 96 Real-Time PCR System cycler with a protocol of 40 °C for 10 min and 95 °C for 5 min, followed by 50 cycles of 95 °C for 10 s and 62 °C for 20 s. Real-time PCR data were visualized through CFX Maestro Software. Primers and probes used are shown in Table 2.

All samples positive in the *T. gondii* real-time PCR after magnetic capture DNA extraction were also analyzed by the same real-time PCR using total DNA from conventional extraction, i.e. the DNA sample also used for detection of *Sarcocystis* spp. This was to compare sensitivity of both methods and to eventually perform a *T. gondii* genotyping in samples of total DNA with Ct values lower than 33, proceeding as previously described (Pardo Gil et al., 2023).

### 2.4 Conventional and real-time PCRs for *Sarcocystis* spp

Conventional PCR was performed on total DNA extracted from each homogenized sample. An initial screening was conducted targeting a ∼ 700 bp fragment of *Sarcocystis* spp. *18S rRNA* gene using primers SarcoF and SarcoR, proceeding according to previous studies (Bentancourt Rossoli et al., 2023; Table 2). PCR runs included a non-template control (ultrapure water), and a positive control (DNA from *S. falcatula*) and were performed in a Mastercycler Nexus Thermal Cycler (Eppendorf SE, Germany) device with a protocol of 94 °C for 15 min, followed by 40 3-step cycles (94 °C 40 s, 59 °C 30 s, 72 °C 1 min), and a final step of 72 °C for 10 min. PCR amplicons were separated and visualized (*E*-Box, Vilber, France) on ethidium bromide-stained 1.5 % agarose gels.

All samples testing positive by the *18S rRNA* PCR (regardless of meat composition) were subjected to a multiplex real-time PCR with specific probes targeting the cattle species *S. hirsuta*, *S. bovifelis/S. rommeli*, *S. cruzi* and *S. ho*min*is*, proceeding essentially as previously reported (Moré et al., 2013). Final reaction volume was 10 μL, of which 2 μL of template DNA, 5 μL of 2× Mastermix (SensiFAST™ Probe NO-ROX Kit; Bioline Meridian Life Science, Memphis, USA), 0.9 μL of nuclease-free water, 1.3 μL of the mix of primers, and 0.8 μL of the mix of TaqMan probes (20 μL TAQ-Probe *S. cruzi*, 40 μL TAQ-Probe *S. hominis*, 40 μL TAQ-Probe *S. hirsuta*, 60 μL TAQ-Probe *S. bovifelis/S. rommeli*, all from 10 μM starting solutions*).* Each PCR routine included a non-template control (2 μL ultrapure water), and a positive control (mix of plasmids containing target genes as previously described (Moré et al., 2013). PCR was executed in the CFX 96 Real-Time PCR System cycler with a protocol of 95 °C for 5 min, followed by 40 cycles at 95 °C for 15 s and 62 °C for 40 s.

To assess the presence of the zoonotic species *S. suihominis*, a conventional PCR targeting a fragment of about 800 bp of the mitochondrial Cytochrome Oxydase Subunit I (*COI*) was performed on all samples containing pork, proceeding as previously described (Moré et al., 2025). The protocol consisted in 95 °C for 15 min, 45 cycles (94 °C for 30 s, 53 °C for 30 s and 72 °C for 90 s), and 72 °C for 10 min. After amplification, the samples were analyzed using the QIAxcel Connect Gel capillary gel electrophoresis system (QIAGEN, Germantown, MD) using the QIAxcel DNA screening cartridge (QIAGEN).

Selected PCR products were submitted for Sanger sequencing: samples positive in the *S. suihominis* specific *COI* mtDNA PCR, and samples positive in the screening *18S* rRNA PCR (excluding beef samples positive in the cattle-specific multiplex real-time PCR, and pork samples analyzed for zoonotic *Sarcocystis* sp. through the *S. suihominis* specific *COI* mtDNA PCR). Mixed-meat samples were not selected for Sanger sequencing.

Primers and probes used are shown in Table 2.

### 2.5 ***Sarcocystis*** spp. **Sanger sequencing**

The amplicons were purified using the Zymoclean Gel DNA Recovery Kit (Zymo Research). Concentrations were assessed using a NanoDrop One spectrophotometer (Thermo Scientific), and samples were diluted as necessary to fall within the optimal range for sequencing (4–17 ng/μL). Purified products and respective PCR primers were submitted to a commercial Sanger sequencing provider (Microsynth, Switzerland). Chromatograms were aligned and analyzed in Geneious Prime (v2023.0.4), and consensus sequences were compared with other sequences available in GenBank using BLAST.

### 2.6 **Detection of *Sarcocystis*** spp. **muscle cysts by microscopy**

From each RTE-meat sample, 5 to 10 g were freed as much as possible from fat and connective tissues and homogenized with 40 mL of Phosphate Buffered Saline (PBS; pH 7.4, Sigma-Aldrich, USA) using a Rotor Memory Blender mixer (Rotor Lips AG, Switzerland). The homogenate was filtered through a tea strainer and subsequently centrifuged at 600 *g* for five minutes. Thereafter, the supernatant was discarded and the resulting pellet resuspended in 20–25 mL of PBS. Three 2 mL aliquots of the suspension were examined under a stereomicroscope (Zeiss Stemi 508) in Petri dishes to detect the presence of *Sarcocystis* muscle cysts at 40× magnification. Structures resembling cysts were isolated, stained with Trypan blue, and observed by light microscopy at 200× magnification. Samples were classified as positive if cysts or cyst fragments were observed; ambiguous or absent findings were classified as negative.

### 2.7 Analysis of associated factors

Factors related to the presence of parasitic DNA were assessed separately for each of the following PCR tests: *T. gondii* (MC-PCR), *Sarcocystis* spp., *S. hominis,* and *S. suihominis*. For *T. gondii* and *Sarcocystis* spp., the same set of binary predictors was investigated: animal species (six binary variables), meat origin (Swiss vs. foreign), product type (salami-like vs. ham-like) and the product labeling (organic vs. non-organic). In addition, two interaction terms (beef x origin and pork x origin) were included as predictor variables (Table 3). Regarding *S. hominis* and *S. suihominis*, only RTE samples containing beef and pork or wild boar, respectively, were considered. The predictor variable for species was replaced by a predictor for meat composition (mixed vs. pure). Aside from this, the same set of predictors was used: meat origin, product type, and product labeling.

**Table 3:** Description of the binary predictor variables and interaction terms.

Variable	Description
Beef	Presence of bovine meat in the product.
Pork	Presence of domestic pig meat in the product.
Horse	Presence of horse meat in the product.
Wild boar	Presence of wild boar meat in the product.
Deer	Presence of deer meat in the product.
Poultry	Presence of chicken or turkey meat in the product.
Origin	Product contains meat of certified Swiss origin or meat from foreign countries.
Product type	Products contains ground meat comprising various muscle cuts (“salami-like”), as opposed to whole cuts from specific muscles (“ham-like”)
Organic	Product is certified organic according to Swiss criteria.
Beef x Origin	Interaction between variables indicating product containing beef meat of Swiss origin.
Pork x Origin	Interaction between variables indicating product containing pork meat of Swiss origin.
Beef x Organic	Interaction between variables indicating organic product containing beef meat.
Pork x Organic	Interaction between variables indicating organic product containing pork meat.
Mixed meat	Product contains meat from more than one species.

Associations between predictor variables and parasite DNA were explored using logistic Least Absolute Shrinkage and Selection Operator (LASSO) regression. All predictor variables were binary, and one sample was excluded because the country of origin of the meat was missing. This method is well suited for variable selection, as it penalizes regression parameters, possibly shrinking them to zero, while minimizing the loss in model fit (James et al., 2021). Two values of the tuning parameter were selected: λ_min_ (minimum mean cross-validation error), and λ_1se_ (most regularized model with cross-validation error within one standard error of λ_min_). Resulting LASSO regression coefficients of significant predictor variables were exponentiated to yield Odds Ratio (OR = e^coef^). Given the exploratory nature of the study, limited sample size, and low frequencies in some categories, the dataset was not split into training and testing subsets. All statistical analyses were performed using R (R Core Team, 2025) with the package glmnet (Friedman et al., 2010).

## 3 Results

### 3.1 Molecular examination

Among the 201 samples analyzed by *T. gondii* magnetic capture and real-time PCR (MC-qPCR), 30 (14.9 %) tested positive, showing Ct values between 32.5 and 41. All samples testing positive for *T. gondii* DNA contained pork: 17 were made of pork only, seven of pork and beef mixture, four of pork and wild boar mixture and two of pork and deer mixture. Eleven conventionally extracted DNAs from these 30 samples were positive in the *T. gondii* real-time PCR with Ct values of 34–40.

A total of 117 samples (58.2 %) were positive for *Sarcocystis* spp. in the conventional screening PCR. *Sarcocystis*-positive samples included pork, beef, horse, wild boar, deer, various mixed-meat combinations, and a poultry-beef mixture. Notably, samples containing only poultry meat all tested negative for *Sarcocystis* spp. Among the different meat types, products containing game meat, i.e. wild boar or deer, showed the highest positivity rate, with all seven samples positive for *Sarcocystis* spp. (100 %), followed by beef (89.1 %) and horse meat (85.7 %). In contrast, pork showed the lowest infection rate (37.5 %).

A summary of the results of molecular analyses performed is shown in Table 4.

**Table 4:** Summary of analyzed meat products (*n* = 201), their composition, and positivity to *T. gondii*, *Sarcocystis* spp., and zoonotic *Sarcocystis* spp. by molecular methods.

Meat composition	#	*T. gondii*	*Sarcocystis* spp.	*S. hominis*	*S. suihominis*
Pork	96	17 (17.7 %)	36 (37.5 %)	0	3
Wildboar & Pork	4	4 (100 %)	4 (100 %)	0	1
Deer & Pork	3	2 (66.7 %)	3 (100 %)	0	0
Beef	55	0 (0 %)	49 (89.1 %)	12	0
Pork & Beef	21	7 (33.3 %)	18 (85.7 %)	11	0
Horse	5	0 (0 %)	4 (80 %)	0	0
Horse & Beef	1	0 (0 %)	1 (100 %)	0	0
Horse & Pork	1	0 (0 %)	1 (100 %)	0	0
Poultry	11	0 (0 %)	0 (0 %)	0	0
Poultry & Beef	4	0 (0 %)	1 (25 %)	1	0
Total	201	30 (14.9 %)	117 (58.2 %)	24 (11.9 %)	4 (2 %)

Out of 81 pure and mixed beef samples, *Sarcocystis* spp. DNA (*18S* rRNA assay) was found in 69 samples (85.1 %). The multiplex real-time PCR with TaqMan probes targeting four *Sarcocystis* species known to infect cattle revealed *S. cruzi* as the most prevalent, with DNA found in 63 of the 69 (91.3 %) positive samples, followed by *S. bovifelis/S. rommeli* (45/69, 65.2 %), *S. hominis* (24/69, 34.7 %), and *S. hirsuta* (17/69, 24.6 %). Mixed infections were frequent (68.1 %, 47/69). When considering all the samples containing beef (*n* = 81), these results correspond to positivity rates of 77.8 % for *S. cruzi*, 55.6 % for *S. bovifelis/S. rommeli*, 29.6 % for *S. hominis*, and 21.0 % for *S. hirsuta*. Only one of 15 poultry samples analyzed tested positive for *Sarcocystis* spp. in *18S* rRNA PCR. This sample also contained beef and the presence of *S. hominis* DNA was confirmed by multiplex real-time PCR assay.

Among 125 pork-containing products, 62 (49.6 %), were positive for *Sarcocystis* spp. by PCR. When limited to pure pork samples (*n* = 96), the prevalence was lowered to 37.5 %.

Species-specific PCR targeting the *S. suihominis COI* gene identified four positive samples: three from pork products (*coppa* and raw ham from Switzerland, and salami from Italy) and one from a wild boar–pork salami from Italy. The four sequences were registered in GenBank with accession numbers: PX377547- PX377550. The three sequences obtained from pork products were 99.9–100 % identical among them (only one SNP in the PX377549) and the fourth sequence, obtained from a pork-wild boar salami (PX377550) was 97.7–97.9 % identical to the other three (16 and 15 SNPs, respectively). Despite these extensive differences, the translated proteins from all four sequences were almost identical among them and the sequence with more SNPs showed only one different amino acid (PX377550). Pure pork samples positive in the initial *18S* rRNA assay and negative in the *COI* mtDNA assay (*n* = 14) were attributed to infection with *S. miescheriana* by considering this the most probable option (Moré et al., 2025).

Among seven samples containing horse meat, six were positive for *Sarcocystis* spp. DNA. These included four pure horse meat products, one mixed with pork, and one mixed with beef. Sequencing of the four pure horse meat samples resulted in short sequences of low quality, and no proper alignment could be achieved. The short fragment sequences (158 to 282 bp) showed 100 % identity and coverage with *S. fayeri* (AB661444.1 among others) and *S. betrami* (MH025635 among others).

Products of organic farming showed a 22.2 % (8/36) positivity for *T. gondii* via MC-qPCR, and 19.4 % (7/36) for zoonotic *Sarcocystis* species, whereas for products not labelled as organic positivity rates were slightly lower (*T. gondii* 22/165, 13.3 %; *S. suihominis, S. hominis* 21/165, 12.7 %). When separating for species, 33.3 % (8/24) of organic products containing pork tested positive for *T. gondii* vs. 21.8 % (22/101) of non-organic products containing pork. Similarly, 15.0 (3/20) of organic products containing beef tested positive for *T. gondii* vs. 6.6 % (4/61) of non-organic products containing beef. Regarding zoonotic sarcocysts, 0 % (0/24) organic products with pork tested positive for *S. suihominis* vs. 4.0 % (4/101) of non-organic products containing pork. Similarly, 35.0 % (7/20) of organic products with beef tested positive for *S. hominis* vs. 28.7 % (17/61) of non-organic products with beef.

### 3.2 Microscopic examination

No cysts were visible by microscopic examination in any of the samples.

### 3.3 Associated factors

For *T. gondii*, six variables were selected in the LASSO regression model (see supplementary file). Pork and salami-like products were the strongest positive predictors, remaining in the model even under the stricter λ_1se_ penalty (pork OR_λ1se_ = 2.9, salami-like OR_λ1se_ = 3.3). In salami-like samples containing pork, *T. gondii* DNA was detected in 26 of 64 samples (40.6 %), compared to only 4 of 61 ham-like pork products (6.6 %). Additional positive predictors (wild boar meat, organic labelled meat) and negative predictors (beef, Swiss meat) were retained only in the less penalized model. Of these predictors wild boar showed the strongest coefficient, whereas the others were weaker (supplementary file).

For *Sarcocystis* spp. DNA, positivity was strongly associated with beef and salami-like products (beef OR_λ1se_ = 4.7, salami-like OR_λ1se_ = 2.8). DNA of *Sarcocystis* spp. was identified in 53 of 64 salami-type samples (82.8 %) versus 10 of 61 ham-type samples (16.4 %). Poultry meat showed strong negative association and was retained in both models (poultry OR_λ1se_ = 0.17). Weaker associations were observed in both models between *Sarcocystis* spp. and Swiss pork and Swiss beef, respectively as negative and positive predictors (Swiss pork OR_λ1se_ = 0.56, Swiss beef OR_λ1se_ = 1.1). The model with lesser penalization also suggested potential positive associations with horse meat and negative with pork.

For zoonotic *S. hominis*, salami-like products, mixed-meat products, and Swiss origin were identified as predictors in the less penalized model. However, no variables were retained in the stricter model. Swiss meat products showed a higher positivity rate (36.7 %, 22/60), compared to meat products of European origin (9.5 %, 2/21).

For *S. suihominis* DNA positivity, results were inconclusive, due to the low number of positive samples (*n* = 4).

## 4 Discussion

This study investigated the presence of the cyst-forming coccidia *Toxoplasma gondii* and *Sarcocystis* spp. in RTE meat products commercially available in Switzerland, using molecular and microscopical methods. These products were selected because they are consumed without cooking, thus they may represent a potential source of infection for consumers.

Studies on protozoa in commercially available RTE-meat products are very limited, and none was so far available for Switzerland. Reported prevalence of *T. gondii* in RTE meat products ranged from 1.5 to 8.8 % in Europe and 15.6 to 17 % in Brazil (Bayarri et al., 2012; Costa et al., 2018; Espindola et al., 2022; Gomez-Samblas et al., 2015; Sroka et al., 2019; Warnekulasuriya et al., 1998). Among the 201 samples analyzed in the present study, *T. gondii* DNA was detected in 14.9 % of products. This positivity rate was higher than that observed in previous studies in Europe, potentially due to the use of the highly sensitive MC-qPCR (Opsteegh et al., 2010) in our study. This method enables the analysis of up to 100 g of sample, compared to only a few hundred milligrams in other protocols, thereby substantially increasing the likelihood of detecting the target DNA. The direct comparison with the total DNA extraction from the homogenized sample confirmed the superior sensitivity: only 11 of the total 30 positive samples in MC-qPCR were also detected with the easier and cheaper DNA extraction method. Total DNA was also extracted to attempt genotyping of *T. gondii*. However, since Ct values in these samples were never lower than 33, genotyping could not be conducted (Pardo Gil et al., 2023).

The *T. gondii* positive products included pure pork products and pork mixes (pork with beef, wild boar or deer). Due to the mixed nature of some of the products, it was not possible to attribute the positivity to one or more of the meat types contained. However, the presence of *T. gondii* DNA in pork-containing products aligns with existing literature identifying it as a significant source of toxoplasmosis, and even as the source of one outbreak (Almeria and Dubey, 2021; Batz et al., 2012; BIOHAZ, E. P. on B. H, et al., 2018). This is consistent with the association between pork and *T. gondii* positivity identified by the LASSO method. Therefore, we considered that the present results may reflect a relatively high level of infection with *T. gondii* in domestic pigs from Swiss and European farms. In Switzerland, seroprevalence of *T. gondii*, was reported to vary between 14 % in finishing pigs and 36 % in mother sows (Berger-Schoch et al., 2011b), which supports this interpretation. As suggested by others, this could be related to the frequent presence of cats, and the consequent environmental contamination with oocysts near pig farms (Berger-Schoch et al., 2011a; Dubey, 2009, Dubey, 2022; Stelzer et al., 2019). Wild boar meat was also retained as a significant variable, though conclusions are limited due to the small sample size (wild boar mixed with pork, *n* = 4). These findings differ from those of previous Swiss studies, which found no (*n* = 0/286) or very low (*n* = 1/151) *T. gondii* prevalence using real-time PCR in wild boar muscle samples, mainly diaphragm (Berger-Schoch et al., 2011a; Moré et al., 2025). It is important to note that none of these studies used the MC-qPCR protocol, which may explain parts of the discrepancy. Additionally, all wild boar-pork RTE products were of the salami-type, and ground meat products were more likely to contain parasite DNA in our study. This increased risk of *T. gondii* and *Sarcocystis* spp. DNA may stem from their composition—ground tissues, potentially from multiple animals—raising the chance of including infected tissue. Additionally, these products may use meat from older animals, which are more likely to have acquired parasitic infections through prolonged environmental exposure (Berger-Schoch et al., 2011a; Dubey, 2022). The same association was already previously detected in lamb minced meat in Canada (Lafrance-Girard et al., 2018).

Notably, all products containing only beef, horse, or poultry tested negative for *T. gondii*. While beef has been considered as source of infection, primarily due to its frequent and raw or rare consumption (Belluco et al., 2018; BIOHAZ, E. P. on B. H, et al., 2018), none of the 55 pure beef samples in the present study tested positive for *T. gondii* DNA. Similarly, no positive findings were observed in products containing only horse or poultry, supporting the lower level of infection and the lesser role as source of infection, particularly of the intensively produced poultry (Almeria and Dubey, 2021; Dubey, 2022). However, sample numbers per species were low in our study, and viability of the parasites was not assessed. Depending on the culinary habits of a given population, the importance of different host species in *T. gondii* transmission to humans may vary.

*Sarcocystis* spp. DNA was detected in 58.2 % of samples, with high infection rates observed in beef, equine, and game products. This is consistent with known endemicity in livestock and results from previous studies, showing prevalence in beef of 64–100 % (Ayazian Mavi et al., 2020; Hoeve-Bakker et al., 2019; Prakas et al., 2020; Rubiola et al., 2021, Rubiola et al., 2024), in horse meat of 35–93 % (Abdel-Gaber et al., 2020) and in game up to 100 % (Guardone et al., 2022). By LASSO regression, beef, and particularly Swiss beef, was identified as a positive predictor in both models, as well as horse meat as a weaker positive predictor in the model with lesser penalization. Positivity rate was lower in pork-only products, with a similar positivity to prevalence studies in Europe using muscles from slaughtered animals (Dubey et al., 2016; Imre et al., 2017). Swiss pork was indeed retained in both LASSO regression models as a negative predictor, though with a relatively weak association. Due to the discrepancy between Swiss pork and Swiss beef, Swiss origin alone was not selected as a predictory variable. On the other hand, poultry resulted as a strong protective variable in LASSO regression for *Sarcocystis* spp. presence, indicating a low level of infection of the animals in their short lifetime.

*Sarcocystis suihominis*, the zoonotic species infecting swine, was found in 3.2 % of swine meat-containing samples from Switzerland and Italy (4/125), including one wild boar-pork salami. All swine-only samples positive for *Sarcocystis* spp. and negative for *S. suihominis* (36/100) were considered to be infected with *S. miescheriana* (Moré et al., 2025). In wild boars, prevalence of *S. miescheriana* is generally high and can reach 97 %, while *S. suihominis* is typically rarer, with prevalence below 2 % in European studies (Calero-Bernal et al., 2016; Coelho et al., 2015; Gazzonis et al., 2019; Moré et al., 2025). This is generally higher than in domestic pigs, where *S. suihominis* was not detected at all in a Romanian study (Imre et al., 2017), as well as in pork samples from USA (Calero-Bernal et al., 2015). In contrast, studies conducted in India, China and Brazil have reported significantly higher positivity rates (Chauhan et al., 2020; da Rosa et al., 2024; Huang et al., 2019). The results of the present study show a relatively high positivity for *S. suihominis* DNA in samples containing only pork (3.1 %, 3/96) sold in Switzerland. Furthermore, they suggest environmental contamination of farms with human feces in the producing countries, and in turn, human infection through consumption of raw pork.

Particularly noteworthy was the detection of zoonotic *S. hominis* DNA in roughly one-third of beef products, which is higher than previous data (Dubey and Rosenthal, 2023). While our analysis did not include *S. heydorni* and *S. sigmoideus*, recent studies suggest they may also have a relatively high prevalence and contribute to human infections (Moniot et al., 2025; Rubiola et al., 2024) and their exclusion may lead to underestimation of potential zoonotic burden in these products. Infection rate for both zoonotic and non-zoonotic species was higher in beef than in pork, possibly due to a higher exposure of cattle to contaminated pastures or water. Furthermore, cattle used for RTE production generally live longer than pigs used for the same purpose (more accumulative exposure). LASSO regression analysis also pointed to a potential association between Swiss beef and positivity to *S. hominis* (36.7 % Swiss samples versus 9.5 % other European countries), even though the association was not retained in the more penalized model, possibly due to the limited sample size. These results suggest a higher exposure in Swiss cattle and raise potential concerns about dietary habits and hygiene standards. Beef is frequently being consumed raw or undercooked (Belluco et al., 2018), thus zoonotic *Sarcocystis* spp. may be transmitted to humans. If human feces containing sporocysts contaminate feed or water, the infection cycle is completed. Environmental contamination may arise from open-air defecation, deficient wastewater management, and following conveying of both animal and human feces in liquid manure. Bovine cysticercosis, which shares a similar fecal-oral transmission route with *S. hominis*, has been associated with several risk factors in a Swiss case control study. These included the location of grazing areas near railway lines or car parks, leisure use of pastures, reliance on purchased roughage, and the hosting of farm events that attract visitors (Flütsch et al., 2008). While unproven, it is plausible that high tourist activity in alpine regions, where cattle are seasonally pastured, could contribute to environmental contamination through inadequate sanitation. The recent detection of *S. suihominis* within the wild population of Swiss wild boars could also bear evidence of this human fecal contamination (Moré et al., 2025).

The detection of parasite DNA in commercial RTE products emphasizes the need for better surveillance and control, as well as for improving sanitation. The role of these products in transmission to humans and animals should be analyzed further. Although an NaCl concentration of 1.3 % or other components of the curing process have been reported to inactivate *T. gondii* (Fredericks et al., 2019; D. E. Hill et al., 2018), evidence from end-product testing remains inconsistent (Bayarri et al., 2012; Genchi et al., 2017; Gomez-Samblas et al., 2015; Herrero et al., 2017). A key limitation of the present study is the inability to assess parasite viability. Mouse bioassay, the gold standard for evaluating *T. gondii* viability, was not employed, whereas Trypan Blue Exclusion Test for *Sarcocystis* spp. was not possible because no typical cysts were detected by stereomicroscopy. This absence of observable cysts may indicate successful inactivation via the curing process. However, it is also plausible that curing of the meat rendered the cysts more fragile, therefore leading to rupture during homogenization, or that the complexity and opacity of the meat matrix hindered microscopic detection. Future studies should consider employing concentration methods to isolate bradyzoites and improve visibility within complex processed meat matrices as well as to attempt molecular viability tests like measuring mRNA.

## 5 Conclusions

This study revealed widespread presence of *Sarcocystis* spp. and *T. gondii* in raw ready-to-eat meat products. Pork products showed the highest positivity rate for *T. gondii*, while beef products had a significantly higher rate for *Sarcocystis* spp., with *S. hominis* detected more frequently in products made from Swiss beef compared to those from other European countries, or to pork products containing *S. suihominis* DNA. Poultry products were less likely to harbor these parasites. These findings highlight frequent transmission between intermediate and definitive hosts, including humans and pets. Contamination of pig and cattle farms with feces from these hosts facilitates completion of the parasites' life cycles. Infection of definitive hosts can occur through consumption of raw or undercooked meat or meat products; however, the epidemiological role of RTE-meat products in the transmission remains unclear.

Currently, there are no regulatory or industrial guidelines in Switzerland, ensuring the inactivation of protozoan parasites in the analyzed products, as regulatory focus is typically on taste and bacterial content. Although our study does not resolve this uncertainty, the relatively high detection rate of parasite DNA reinforces the need for further investigation and supports at least a potential role of RTE meat as a source of infection. Development of standardized curing guidelines is warranted to reduce consumer risk. Additionally, consumers and private producers should be educated on the existence of these parasites, the limitations of the curing processes as a protective measure, and effective risk-reduction measures, such as freezing meat prior to preparation, to ensure product safety.

## Funding source declaration

This study was financially supported by the Swiss 10.13039/501100006454Federal Food Safety and Veterinary Office (FSVO), Switzerland. Contract number: 714003117. The funding source was not involved in the study design, collection, analysis and interpretation of data, writing of the report, or decision to submit the article for publication.

## CRediT authorship contribution statement

**Z. Medici:** Writing – review & editing, Writing – original draft, Visualization, Validation, Methodology, Investigation, Formal analysis, Data curation. **N. Marreros:** Writing – review & editing, Formal analysis, Data curation. **S. Molteni:** Writing – review & editing, Investigation. **M.C. Ferreira de Sousa:** Writing – review & editing, Resources. **W. Basso:** Writing – review & editing, Resources. **G. Moré:** Writing – review & editing, Supervision, Resources, Methodology, Data curation, Conceptualization. **C.F. Frey:** Writing – review & editing, Supervision, Resources, Project administration, Methodology, Funding acquisition, Conceptualization.

## Declaration of competing interest

The authors declare that they have no known competing financial interests or personal relationships that could have appeared to influence the work reported in this paper.

## Appendix A Supplementary data

Supplementary material Image 1

## References

[bb0005] Abdel-Gaber R., Al Quraishy S., Dkhil M.A., Alghamdi J., Al-Shaebi E. (2020). Molecular phylogeny of *Sarcocystis fayeri* (Apicomplexa: Sarcocystidae) from the domestic horse *Equus caballus* based on 18S rRNA gene sequences and its prevalence. Lett. Appl. Microbiol..

[bb0010] Almeria S., Dubey J.P. (2021). Foodborne transmission of *toxoplasma gondii* infection in the last decade. An overview. Res. Vet. Sci..

[bb0015] Ayazian Mavi S., Teimouri A., Mohebali M., Sharifi Yazdi M.K., Shojaee S., Rezaian M., Salimi M., Keshavarz H. (2020). *Sarcocystis* infection in beef and industrial raw beef burgers from butcheries and retail stores: A molecular microscopic study. Heliyon.

[bb0020] Basso W., Sollberger E., Schares G., Küker S., Ardüser F., Moore-Jones G., Zanolari P. (2020). *Toxoplasma gondii* and *Neospora caninum* infections in south American camelids in Switzerland and assessment of serological tests for diagnosis. Parasit. Vectors.

[bb0025] Basso W., Holenweger F., Schares G., Müller N., Campero L.M., Ardüser F., Moore-Jones G., Frey C.F., Zanolari P. (2022). *Toxoplasma gondii* and *Neospora caninum* infections in sheep and goats in Switzerland: Seroprevalence and occurrence in aborted foetuses. Food Waterborne Parasitol..

[bb0030] Batz M.B., Hoffmann S., Morris J.G. (2012). Ranking the disease burden of 14 pathogens in food sources in the United States using attribution data from outbreak investigations and expert elicitation. J. Food Prot..

[bb0035] Bayarri S., Gracia M.J., Pérez-Arquillué C., Lázaro R., Herrera A. (2012). *Toxoplasma gondii* in commercially available pork meat and cured ham: A contribution to risk assessment for consumers. J. Food Prot..

[bb0040] Belluco S., Simonato G., Mancin M., Pietrobelli M., Ricci A. (2018). *Toxoplasma gondii* infection and food consumption: A systematic review and meta-analysis of case-controlled studies. Crit. Rev. Food Sci. Nutr..

[bb0045] Bentancourt Rossoli J.V., Moré G., Soto-Cabrera A., Moore D.P., Morrell E.L., Pedrana J., Scioli M.V., Campero L.M., Basso W., Hecker Y.P., Scioscia N.P. (2023). Identification of *Sarcocystis* spp. in synanthropic (Muridae) and wild (Cricetidae) rodents from Argentina. Parasitol. Res..

[bb0050] Berger-Schoch A.E., Herrmann D.C., Schares G., Müller N., Bernet D., Gottstein B., Frey C.F. (2011). Prevalence and genotypes of *toxoplasma gondii* in feline faeces (oocysts) and meat from sheep, cattle and pigs in Switzerland. Vet. Parasitol..

[bb0055] Berger-Schoch A.E., Bernet D., Doherr M.G., Gottstein B., Frey C.F. (2011). *Toxoplasma gondii* in Switzerland: a serosurvey based on meat juice analysis of slaughtered pigs, wild boar, sheep and cattle. Zoonoses Public Health.

[bb0060] BIOHAZ, E. P. on B. H, Koutsoumanis K., Allende A., Alvarez-Ordóñez A., Bolton D., Bover-Cid S., Chemaly M., Davies R., De Cesare A., Herman L., Hilbert F., Lindqvist R., Nauta M., Peixe L., Ru G., Simmons M., Skandamis P., Suffredini E., Cacciò S., Robertson L. (2018). Public health risks associated with food-borne parasites. EFSA J..

[bb0065] Calero-Bernal R., Verma S.K., Oliveria S., Yang Y., Rosenthal B.M., Dubey J.P. (2015). In the United States, negligible rates of zoonotic sarcocystosis occur in feral swine that, by contrast, frequently harbour infections with *Sarcocystis miescheriana*, a related parasite contracted from canids. Parasitology.

[bb0070] Calero-Bernal R., Pérez-Martín J.E., Reina D., Serrano F.J., Frontera E., Fuentes I., Dubey J.P. (2016). Detection of zoonotic Protozoa *toxoplasma gondii* and *Sarcocystis suihominis* in wild boars from Spain. Zoonoses Public Health.

[bb0075] Chauhan R.P., Kumari A., Nehra A.K., Ram H., Garg R., Banerjee P.S., Karikalan M., Sharma A.K. (2020). Genetic characterization and phylogenetic analysis of *Sarcocystis suihominis* infecting domestic pigs (*Sus scrofa*) in India. Parasitol. Res..

[bb0080] Coelho C., Gomes J., Inácio J., Amaro A., Mesquita J.R., Pires I., Lopes A.P., Vieira-Pinto M. (2015). Unraveling *Sarcocystis miescheriana* and *Sarcocystis suihominis* infections in wild boar. Vet. Parasitol..

[bb0085] Cook A.J.C., Holliman R., Gilbert R.E., Buffolano W., Zufferey J., Petersen E., Jenum P.A., Foulon W., Semprini A.E., Dunn D.T. (2000). Sources of *toxoplasma* infection in pregnant women: European multicentre case-control study. European research network on congenital toxoplasmosis. BMJ.

[bb0090] Costa D.F., Fowler F., Silveira C., Nóbrega M.J., Nobrega H.A.J., Nascimento H., Rizzo L.V., Commodaro A.G., Belfort R. (2018). Prevalence of *toxoplasma gondii* DNA in processed pork meat. Foodborne Pathog. Dis..

[bb0095] da Rosa G., Roman I.J., Gressler L.T., Cargnelutti J.F., Vogel F.S.F. (2024). Molecular identification of *Sarcocystis* species in wild boar (*Sus scrofa*) and pigs (*Sus scrofa domesticus*) in Brazil. Vet. Parasitol..

[bb0100] Devleesschauwer B., Bouwknegt M., Dorny P., Gabriël S., Havelaar A.H., Quoilin S., Robertson L.J., Speybroeck N., Torgerson P.R., van der Giessen J.W.B., Trevisan C. (2017). Risk ranking of foodborne parasites: state of the art. Food Waterborne Parasitol..

[bb0105] Dubey J.P. (2009). Toxoplasmosis in pigs—the last 20 years. Vet. Parasitol..

[bb0110] Dubey J.P. (2015). Foodborne and waterborne zoonotic sarcocystosis. Food Waterborne Parasitol..

[bb0115] Dubey J.P. (2021). Outbreaks of clinical toxoplasmosis in humans: five decades of personal experience, perspectives and lessons learned. Parasit. Vectors.

[bb0120] Dubey J.P. (2022).

[bb0125] Dubey J.P., Rosenthal B.M. (2023). Bovine sarcocystosis: Sarcocystis species, diagnosis, prevalence, economic and public health considerations, and association of *Sarcocystis* species with eosinophilic myositis in cattle. Int. J. Parasitol..

[bb0130] Dubey J.P., Calero-Bernal R., Rosenthal B.M., Speer C.A., Fayer R. (2016).

[bb0135] Espindola B.D., Fernandes F.D., Roman I.J., Samoel G.V.A., Barcelos R.A.D., Döhler A.R., Botton S.Á., Vogel F.S.F., Sangioni L.A. (2022). Detection of *Sarcocystis* spp. and *toxoplasma gondii* in swine and detection of DNA of these protozoa in tissues and sausages. Rev. Bras. Parasitol. Vet..

[bb0140] Fayer R., Esposito D.H., Dubey J.P. (2015). Human infections with *Sarcocystis* species. Clin. Microbiol. Rev..

[bb0145] Flütsch F., Heinzmann D., Mathis A., Hertzberg H., Stephan R., Deplazes P. (2008). Case-control study to identify risk factors for bovine cysticercosis on farms in Switzerland. Parasitology.

[bb0150] Fredericks J., Hawkins-Cooper D.S., Hill D.E., Luchansky J., Porto-Fett A., Gamble H.R., Fournet V.M., Urban J.F., Holley R., Dubey J.P. (2019). Low salt exposure results in inactivation of *toxoplasma gondii* bradyzoites during formulation of dry cured ready-to-eat pork sausage. Food Waterborne Parasitol..

[bb0155] Friedman J., Hastie T., Tibshirani R. (2010). Glmnet: regularization paths for generalized linear models via coordinate descent. J. Stat. Softw..

[bb0160] Gazzonis A.L., Gjerde B., Villa L., Minazzi S., Zanzani S.A., Riccaboni P., Sironi G., Manfredi M.T. (2019). Prevalence and molecular characterisation of *Sarcocystis miescheriana* and *Sarcocystis suihominis* in wild boars (*Sus scrofa*) in Italy. Parasitol. Res..

[bb0165] Genchi M., Vismarra A., Mangia C., Faccini S., Vicari N., Rigamonti S., Prati P., Marino A.M., Kramer L., Fabbi M. (2017). Lack of viable parasites in cured ‘Parma ham’ (PDO), following experimental *toxoplasma gondii* infection of pigs. Food Microbiol..

[bb0170] Gomez-Samblas M., Vílchez S., Racero J.C., Fuentes M.V., Osuna A. (2015). Quantification and viability assays of *toxoplasma gondii* in commercial “Serrano” ham samples using magnetic capture real-time qPCR and bioassay techniques. Food Microbiol..

[bb0175] Guardone L., Armani A., Mancianti F., Ferroglio E. (2022). A review on *Alaria alata, toxoplasma gondii* and *Sarcocystis* spp. in mammalian game meat consumed in Europe: epidemiology, risk management and future directions. Animals.

[bb0180] Herrero L., Gracia M.J., Pérez-Arquillué C., Lázaro R., Herrera A., Bayarri S. (2017). *Toxoplasma gondii* in raw and dry-cured ham: the influence of the curing process. Food Microbiol..

[bb0185] Hill D., Dubey J.P. (2002). *Toxoplasma gondii*: transmission, diagnosis and prevention. Clin. Microbiol. Infect..

[bb0190] Hill D.E., Luchansky J., Porto-Fett A., Gamble H.R., Fournet V.M., Hawkins-Cooper D.S., Urban J.F., Gajadhar A.A., Holley R., Juneja V.K., Dubey J.P. (2018). Rapid inactivation of *toxoplasma gondii* bradyzoites during formulation of dry cured ready-to-eat pork sausage. Food Waterborne Parasitol..

[bb0195] Hoeve-Bakker B.J.A., van der Giessen J.W.B., Franssen F.F.J. (2019). Molecular identification targeting cox1 and 18S genes confirms the high prevalence of *Sarcocystis* spp. in cattle in the Netherlands. Int. J. Parasitol..

[bb0200] Huang Z., Ye Y., Zhang H. (2019). Morphological and molecular characterizations of *Sarcocystis miescheriana* and *Sarcocystis suihominis* in domestic pigs (*Sus scrofa*) in China. Parasitol. Res..

[bb0205] Imre K., Sala C., Morar A., Imre M., Ciontu C., Chisăliță I., Dudu A., Matei M., Dărăbuș G. (2017). Occurrence and first molecular characterization of *Sarcocystis* spp. in wild boars (*Sus scrofa*) and domestic pigs (*Sus scrofa domesticus*) in Romania: public health significance of the isolates. Acta Trop..

[bb0210] James G., Witten D., Hastie T., Tibshirani R. (2021).

[bb0215] Lafrance-Girard C., Arsenault J., Thibodeau A., Opsteegh M., Avery B., Quessy S. (2018). *Toxoplasma gondii* in retail beef, lamb, and pork in Canada: prevalence, quantification, and risk factors from a public health perspective. Foodborne Pathog. Dis..

[bb0220] Moniot M., Combes P., Costa D., Argy N., Durieux M.F., Nicol T., Nourrisson C., Poirier P. (2025). Simultaneous detection of *Sarcocystis hominis, S. Heydorni*, and *S. Sigmoideus* in human intestinal Sarcocystosis, France, 2021–2024. Emerg. Infect. Dis..

[bb0225] Moré G., Abrahamovich P., Jurado S., Bacigalupe D., Marin J.C., Rambeaud M., Venturini L., Venturini M.C. (2011). Prevalence of *Sarcocystis* spp. in Argentinean cattle. Vet. Parasitol..

[bb0230] Moré G., Schares S., Maksimov A., Conraths F.J., Venturini M.C., Schares G. (2013). Development of a multiplex real time PCR to differentiate *Sarcocystis* spp. affecting cattle. Vet. Parasitol..

[bb0235] Moré G., Filippini C., Oehm A.W., Ruetten M., Hemphill A., Frey C.F., Basso W. (2025). *Sarcocystis* spp. and *toxoplasma gondii* in muscles from wild boars (*Sus scrofa*) consumed in Switzerland. Int. J. Parasitol..

[bb0240] Opsteegh M., Langelaar M., Sprong H., den Hartog L., De Craeye S., Bokken G., Ajzenberg D., Kijlstra A., der Giessen J., van. (2010). Direct detection and genotyping of *toxoplasma gondii* in meat samples using magnetic capture and PCR. Int. J. Food Microbiol..

[bb0245] Pardo Gil M., Hegglin D., Briner T., Ruetten M., Müller N., Moré G., Frey C.F., Deplazes P., Basso W. (2023). High prevalence rates of *toxoplasma gondii* in cat-hunted small mammals - evidence for parasite induced behavioural manipulation in the natural environment?. Int. J. Parasitol..

[bb0250] Pleyer U., Schlüter D., Mänz M. (2014). Ocular toxoplasmosis: recent aspects of pathophysiology and clinical implications. Ophthalmic Res..

[bb0255] Poulsen C.S., Stensvold C.R. (2014). Current status of epidemiology and diagnosis of human Sarcocystosis. J. Clin. Microbiol..

[bb0260] Prakas P., Strazdaitė-Žielienė Ž., Januškevičius V., Chiesa F., Baranauskaitė A., Rudaitytė-Lukošienė E., Servienė E., Petkevičius S., Butkauskas D. (2020). Molecular identification of four *Sarcocystis* species in cattle from Lithuania, including *S. Hominis*, and development of a rapid molecular detection method. Parasit. Vectors.

[bb0265] R Core Team (2025). https://www.R-project.org/.

[bb0270] Reischl U., Bretagne S., Krüger D., Ernault P., Costa J.-M. (2003). Comparison of two DNA targets for the diagnosis of toxoplasmosis by real-time PCR using fluorescence resonance energy transfer hybridization probes. BMC Infect. Dis..

[bb0275] Rosenthal B.M. (2021). Zoonotic Sarcocystis. Res. Vet. Sci..

[bb0280] Rubiola S., Civera T., Panebianco F., Vercellino D., Chiesa F. (2021). Molecular detection of cattle *Sarcocystis* spp. in north-West Italy highlights their association with bovine eosinophilic myositis. Parasit. Vectors.

[bb0285] Rubiola S., Moré G., Civera T., Hemphill A., Frey C.F., Basso W., Colasanto I., Vercellino D., Fidelio M., Lovisone M., Chiesa F. (2024). Detection of *Sarcocystis hominis, Sarcocystis bovifelis, Sarcocystis cruzi, Sarcocystis hirsuta* and *Sarcocystis sigmoideus* sp. nov. in carcasses affected by bovine eosinophilic myositis. *Food and waterborne*. Parasitology.

[bb0290] Sobanski V., Ajzenberg D., Delhaes L., Bautin N., Just N. (2013). Severe toxoplasmosis in immunocompetent hosts: be aware of atypical strains. Am. J. Respir. Crit. Care Med..

[bb0295] Sroka J., Bilska-Zajac E., Wójcik-Fatla A., Zajac V., Dutkiewicz J., Karamon J., Piotrowska W., Cencek T. (2019). Detection and molecular characteristics of *toxoplasma gondii* DNA in retail raw meat products in Poland. Foodborne Pathog. Dis..

[bb0300] Stelzer S., Basso W., Benavides Silván J., Ortega-Mora L.M., Maksimov P., Gethmann J., Conraths F.J., Schares G. (2019). *Toxoplasma gondii* infection and toxoplasmosis in farm animals: risk factors and economic impact. Food Waterborne Parasitol..

[bb0305] Verordnung des EDI über die Hygiene beim Schlachten (VHyS) (2006). https://www.fedlex.admin.ch/eli/cc/2005/816/de.

[bb0310] Warnekulasuriya M.R., Johnson J.D., Holliman R.E. (1998). Detection of *toxoplasma gondii* in cured meats. Int. J. Food Microbiol..

