# Supplementary material for: High frequency of DNA detection of toxoplasma gondii and zoonotic Sarcocystis spp. in ready-to-eat meat products purchased in Switzerland
Source: Food Waterborne Parasitol. 2025 Nov 12;41:e00301. doi: 10.1016/j.fawpar.2025.e00301 (PMC12663679; doi:10.1016/j.fawpar.2025.e00301)
Supplement: Supplementary file 1 — Supplementary material [file mmc1.docx]

**High frequency of detection of *Toxoplasma gondii* and zoonotic *Sarcocystis* spp*.* in ready-to-eat meat products purchased in Switzerland**

Medici Z.1, Marreros N.2, Molteni S.3, Ferreira de Sousa MC.1, Basso W.1, Moré G..1, Frey C.F.1

1Institute of Parasitology, Department of Infectious Diseases and Pathobiology, Vetsuisse Faculty, University of Bern, Länggassstrasse 122, 3012 Bern, Switzerland

2School of Agricultural, Forest and Food Sciences HAFL, Bern University of Applied Sciences, Länggasse 85, 3052, Zollikofen, Switzerland

3 Department of Veterinary Medicine and Animal Sciences (DIVAS), University of Milan, Via dell’Università 6, 26900 Lodi, Italy

**Supplementary material A**

Cross-validation curve, coefficient path and parameter estimates for each logistic LASSO regression. Two values of the tuning parameter were selected: λ_min_ which minimizes cross-validation error and λ_1se_ which selects the smallest model for which the cross-validation error is within one standard error of the model with λ_min._

Fig A1: Cross-validation curve (left) and coefficients path (right) for the LASSO model to *T. gondii*. The vertical dotted lines display values of λ_min_ and λ_1se_ on a log scale .


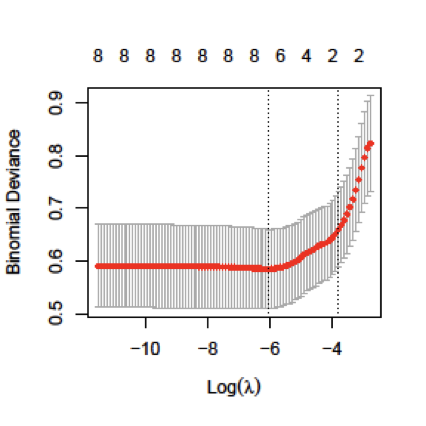

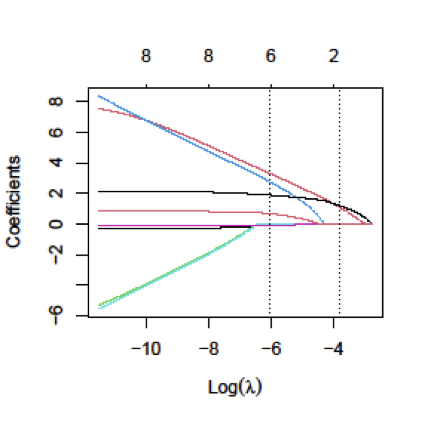


Table A1: Parameter estimates for the logistic LASSO regression when assessing predictor variables against *T. gondii*. Both sets of parameters are shown, at λ_min_ and λ_1se_.

| Parameter name | λ_min_ | λ_1se_ |
| --- | --- | --- |
| Beef | -0. 08 | - |
| Pork | 3.29 | 1.06 |
| Horse | - | - |
| Wild boar | 2.75 | - |
| Deer | - | - |
| Poultry | - | - |
| Swiss | -0.10 | - |
| Salami-type | 1.89 | 1.20 |
| Organic | 0.68 | - |
| Beef x Swiss | - | - |
| Pork x Swiss | - | - |

Fig A2: Cross-validation curve (left) and coefficients path (right) for the LASSO model to *Sarcocystis* sp. The vertical dotted lines display values of λ_min_ and λ_1se_ on a log scale.


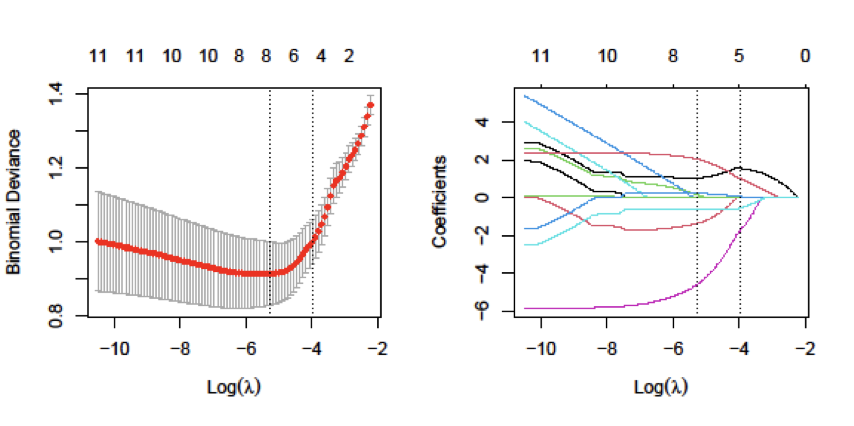


Table A2: Parameter estimates for the logistic LASSO regression when assessing predictor variables against *Sarcocystis sp.* Both sets of parameters are shown, at λ_min_ and λ_1se_.

| Parameter name | λ_min_ | λ_1se_ |
| --- | --- | --- |
| Beef | 1.05 | 1.54 |
| Pork | -1.36 | - |
| Horse | 0.17 | - |
| Wild boar | - | - |
| Deer | - | - |
| Poultry | -4.59 | -1.76 |
| Swiss | - | - |
| Salami-type | 2.04 | 1.02 |
| Organic | - | - |
| Beef x Swiss | 0.22 | 0.06 |
| Pork x Swiss | -0.60 | -0.57 |

Fig A3: Cross-validation curve (left) and coefficients path (right) for the LASSO model to *Sarcocystis* *hominis*. The vertical dotted lines display values of λ_min_ and λ_1se_ on a log scale.


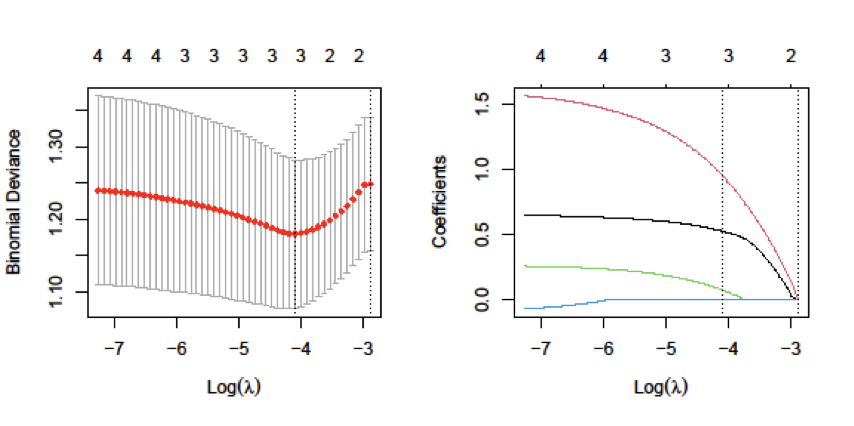


Table A3: Parameter estimates for the logistic LASSO regression when assessing predictor variables against *Sarcocystis hominis.* Both sets of parameters are shown, at λ_min_ and λ_1se_.

| Parameter name | λ_min_ | λ_1se_ |
| --- | --- | --- |
| Mixed Beef | 0.53 | - |
| Swiss | 0.95 | - |
| Salami-type | 0.08 | - |
| Organic | - | - |

Fig A4: Cross-validation curve (left) and coefficients path (right) for the LASSO model to *Sarcocystis* *suihominis*. Both tuning parameters λ_min_ and λ_1se_ overlap.


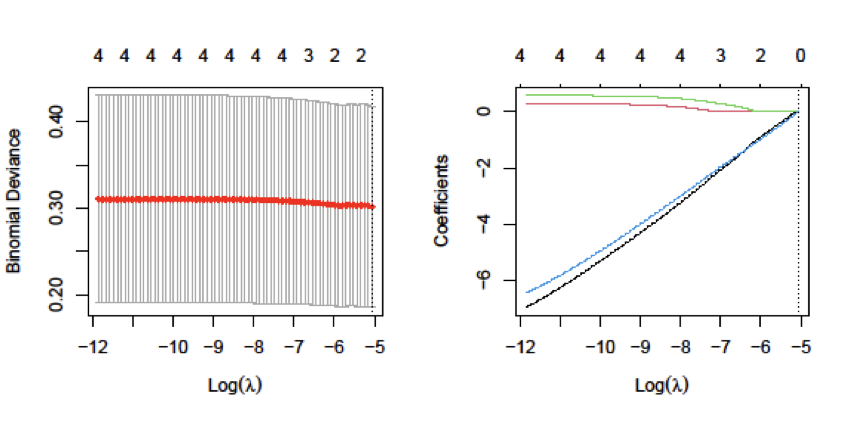


Table A4: Parameter estimates for the logistic LASSO regression when assessing predictor variables against *Sarcocystis suihominis.* Both sets of parameters are shown, at λ_min_ and λ_1se_.

| Parameter name | λ_min_ | λ_1se_ |
| --- | --- | --- |
| Mixed Pork | - | - |
| Swiss | - | - |
| Salami-type | - | - |
| Organic | - | - |
